# Locoregional Anaesthesia for Laparotomy: A Literature Review and Subsequent Case Series Highlighting the Potential of an Alternative Anaesthetic Technique

**DOI:** 10.7759/cureus.45529

**Published:** 2023-09-19

**Authors:** Calvin Coe, Paul W Shuttleworth, Deepak Rangappa, Mostafa Abdel-Halim

**Affiliations:** 1 General Surgery, Tameside General Hospital, Manchester, GBR; 2 Anaesthesia, Tameside General Hospital, Manchester, GBR

**Keywords:** epidural anaesthesia, spinal anaesthesia, locoregional anesthesia, awake laparotomy, laparotomy decision

## Abstract

Laparotomy is a surgical incision utilised in both emergency and elective scenarios to gain access to abdominal surgery. General anaesthesia is usually necessitated due to the substantial insult of the approach and to facilitate organ relaxation and paralysis. However, this brings with it the need for an assessment of the suitability of the anaesthetic technique, with a large number of patients having comorbidities significant enough to exclude them from surgery. Locoregional anaesthesia, provided via spinal, epidural, or a combined approach offers a means of providing anaesthesia that places a reduced level of strain on patients’ cardiorespiratory function. We review the existing literature on the topic of so-called "awake laparotomy" performed with locoregional anaesthesia and present a case series including both elective and emergency procedures.

## Introduction

Laparotomy is the surgical incision commonly used to obtain access to the peritoneal cavity. It has a wide range of indications and is utilised for both emergency and elective procedures. An estimated 40,000 laparotomies are performed annually in the United Kingdom [1]. Whilst this number has fallen with the expansion of minimally invasive surgery, the incision will remain an essential technique in the surgeon’s toolbox. Emergencies with significant intraperitoneal contamination, or penetrating or blunt trauma, necessitate a laparotomy as the incision of choice to allow for full abdominal access. It is also used in cases where there are significant abdominal adhesions, or grossly distended bowel, both of which can make a minimal access approach unfeasible.

Entry into the peritoneal cavity during intraabdominal surgery brings with it a tremendous physiological insult, with the possibility of major systemic complications and death in even the fittest patients. The impact of general anaesthesia physiologically on the cardiorespiratory system, in particular, can further increase morbidity and mortality risk, particularly in patients with underlying disease. Therefore, a decision to undertake a laparotomy in the comorbid with a poor functional baseline, or systematically unwell patient, requires full anaesthetic assessment with risk analysis to enable the surgical and anaesthetic teams to decide whether operating is appropriate and to inform the patient accordingly. A variety of scoring systems are available to assist in this process, with some of the more commonly adopted being the Portsmouth physiologic and operative severity score for the study of mortality and morbidity (P-POSSUM) [2], American College of Surgeons National Surgical Quality Improvement Program (ACS-NQISP) [3], and the Charleston Comorbidity Index 2 [4].

If, when considering the results of these scoring systems, the anaesthetic risk is deemed too high, and if the patient is not suitable to undergo general anaesthesia, alternative options are limited. Patients necessitating laparotomy due to an urgent scenario or contraindications to a minimal access approach for a less pressing procedure may be excluded from potentially life-saving operations due to unsuitability for general anaesthesia, without an alternative.

We review the literature on regional anaesthesia for laparotomy, specifically via spinal, epidural, or a combined approach to establish the existing evidence base and present a case series of laparotomies performed under combined epidural and spinal anaesthesia in a UK District General hospital to add support to this approach as consideration for specific patient subgroups.

## Case presentation

Case series 

We present a case series from a UK District General Hospital of three laparotomies performed in patients considered to be high risk for general anaesthesia; all were carried out under combined spinal and epidural anaesthesia. Each resulted in a successful outcome for the patient involved, with no patients requiring conversion to general anaesthesia. All cases included in this review were discussed at the local governance meeting, and consent for publication with the inclusion of anonymised radiological images was obtained. 

Case 1

A 75-year-old male, with a background of hypertension, previous myocardial infarction, chronic obstructive pulmonary disease, and pulmonary fibrosis, was admitted with severe abdominal pain and bilious vomiting. He had recently completely radical chemoradiotherapy for a junctional oesophagogastric cancer, with an indwelling gastrostomy tube for feeding. The abdomen was distended and tender on palpation; however, no signs of peritonitis were present. Blood tests on admission were unremarkable, with only a mild rise in the inflammatory markers, normal lactate, amylase, and renal function.

**Figure 1:**
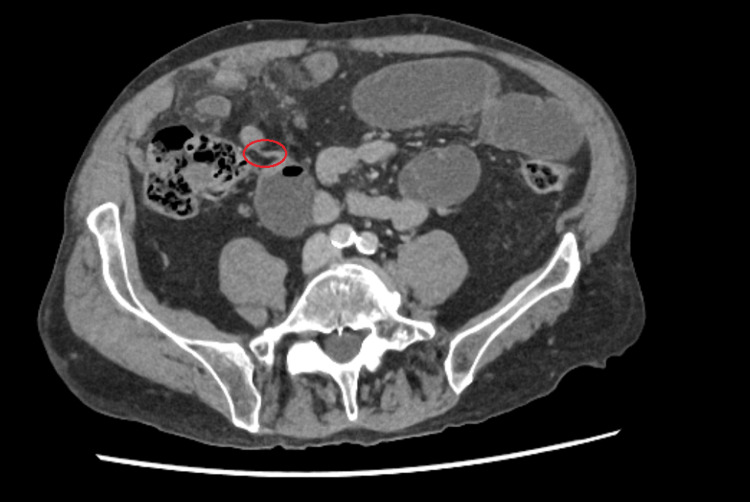
Portal venous phase CT abdomen – axial view demonstrating small bowel obstruction with jejunal and ileal dilation and the transition point

A CT scan of the abdomen and pelvis showed a picture in keeping with small bowel obstruction with two transition points identified likely due to adhesive bands: one within the mid ileum and another within the terminal ileum. This was associated with mesenteric oedema and thickening raising concern about the possibility of closed-loop obstruction (Figure 1).

An anaesthetic assessment was carried out, and the patient was identified as ASA grade III. A P-POSSUM score [2] was calculated, revealing an 11% risk of mortality and 85% risk of morbidity from laparotomy. Given the patient’s significant underlying respiratory disease, his fitness for general anaesthesia was markedly affected, and it was therefore decided to operate under combined spinal and epidural anaesthesia. This was performed via a two-needle technique, with the spinal block performed at L3-L4 and the epidural catheter sited at T10. This allowed for a sensory block to be established at the level of T6 and a motor block at T7, to avoid intercostal muscle compromise. Additionally, 2.4 mL of 0.5% heavy bupivacaine and 500 mcg diamorphine were injected intrathecally, alongside two intra-operative top-up doses of 5 mL of 0.5% levobupivacaine via epidural catheter due to the operation length. An infusion of 10 mL/hour of 0.1% bupivacaine and 2 mcg/mL of fentanyl was established via the epidural route for postoperative pain, and 1 mg of midazolam IV was provided for sedation. No intra-operative inotropic support was required.

The surgery involved a small central midline laparotomy incision. Adhesions causing closed-loop obstruction were found and released effectively without the need for bowel resection as the entrapped small bowel loop was found to be viable. The patient’s postoperative course was uneventful, and they were discharged on the eighth postoperative day.

Case 2

A 78-year-old female, with known atrial fibrillation, congestive cardiac failure, hypothyroidism, and previous breast cancer, presented with a history of persistent abdominal pain and bilious vomiting. Her past surgical history included previous laparotomy and abdominal aortic aneurysm repair several years earlier. Along with anti-hypertensives and diuretics, the patient was also on the direct oral anticoagulant apixaban regularly, which was held prior to surgery. Clinical examination revealed a mildly distended and tender abdomen, but no signs of peritonitis. She had, however, presented two days after the onset of her symptoms and appeared to have aspiration pneumonia with hypoxia, tachypnoea, and lung shadowing on chest x-ray.

**Figure 2:**
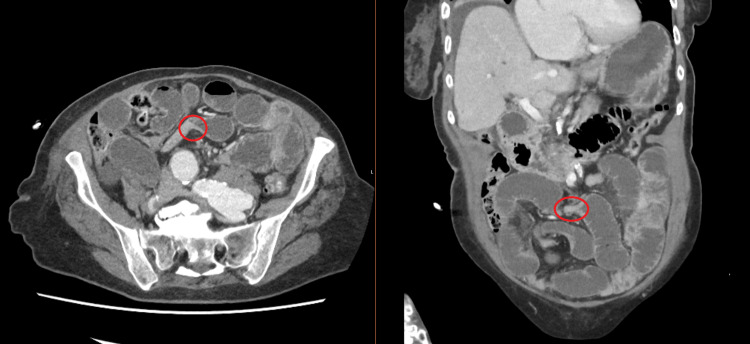
Axial and coronal portal venous phase CT images demonstrating a high-grade small bowel obstruction, with a transition point noted in the right iliac fossa

Blood tests taken on admission showed a mildly raised white cell count, but all other values were within normal limits, including haemoglobin concentration, platelet count, and serum lactate. A CT scan of the abdomen and pelvis revealed features of a high-grade, small-bowel obstruction, with multiple distended loops of proximal small bowel, and an abrupt transition in the region of the proximal to mid ileum, within the right iliac fossa. The appearances were in keeping with adhesional obstruction (Figure 2).

She was initially treated conservatively with IV fluid resuscitation and electrolyte replacement alongside nasogastric tube decompression. This continued for three days, with close monitoring of her clinical and biochemical markers. Due to her ongoing chest sepsis during this period, she was not deemed fit for definitive surgical management. She remained stable but without resolution of the obstruction; therefore, the decision was made to proceed with surgery at this point.

The patient’s ASA grade was calculated to be II, but in view of the clinical and radiological evidence of an established chest infection, her risks from general anaesthesia were deemed to be too high. Her P-POSSUM peri-operative risk calculation [2] estimated a 12.6% mortality risk. The decision was, therefore, made to proceed with surgery under regional anaesthesia alone; the use of apixaban was not regarded to be a contraindication given the normal haemoglobin and platelet count and considering it had been held pre-operatively.

As per the previous case, L3-L4 was the site of spinal needle insertion, with a separate epidural catheter sited at T10 to avoid cardiac sympathetic blockade. The agents used were the same, albeit with doses altered due to patient weight; 2.2 mL of 0.5% heavy bupivacaine and 400 mcg of diamorphine intrathecally, alongside a single bolus of 5 mL of 0.5% levobupivacaine via the epidural catheter. The same epidural infusion was set up, utilizing 0.125% bupivacaine and 2 mcg/mL fentanyl. Arterial line insertion was performed to allow for continuous monitoring.

A midline laparotomy was performed, and a tight adhesional band along with omental adhesions were found to be the culprit for obstruction. This had caused a gangrenous constriction ring in the small bowel, but without perforation or contamination. Division of the band was performed, and limited resection of the gangrenous small bowel was carried out with end-to-end hand-sewn anastomosis.

The patient remained awake throughout the procedure, with no additional sedation required. They remained physiologically stable throughout, albeit with a moderate drop in mean arterial pressure; this was addressed with a single 0.5 mL bolus of 150 mcg/mL metaraminol intra-operatively, alongside a continuous infusion of 500 mcg/mL at 2 mL/hour. The patient was transferred to a high dependency unit (HDU) post-operatively, where vasopressors were continued alongside intravenous antibiotics and the epidural infusion at approximately 6-8 mL/hour. No respiratory support was required. After two days of epidural analgesia, the patient was stepped down to patient-controlled intravenous morphine infusion. Her postoperative course was without complication, with a successful discharge home on day nine post-op.

Case 3

**Figure 3:**
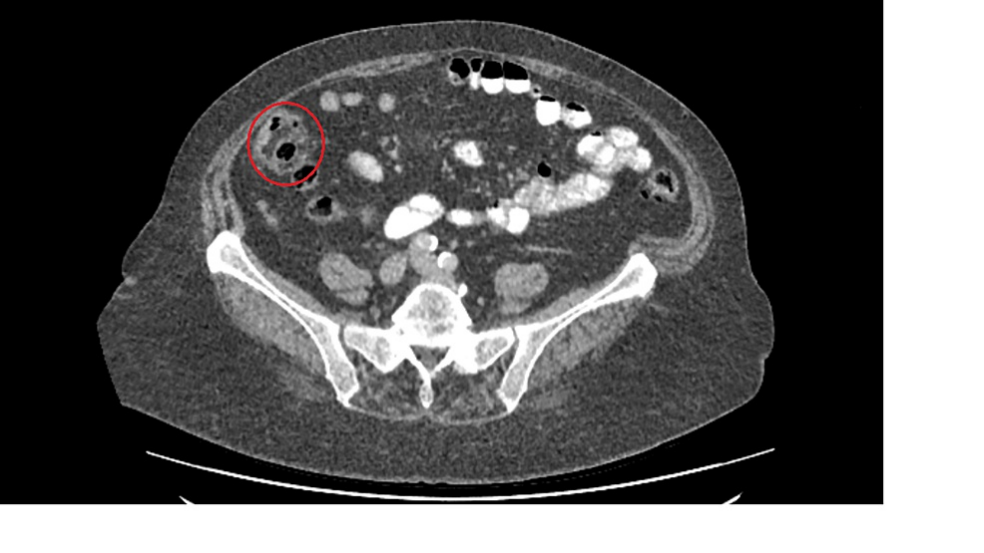
Portal venous phase CT abdomen - axial view showing circumferential caecal wall thickening

An 80-year-old female patient was investigated for anaemia and found to have caecal adenocarcinoma. A staging CT scan confirmed no distant metastasis with locoregional lymphadenopathy. Radiological TNM staging was T2 N1 M0 (Figure 3).

She had multiple significant comorbidities, including ischaemic heart disease, atrial fibrillation with an indwelling pacemaker, and systemic and pulmonary hypertension (cardiac catheterisation demonstrated a pulmonary artery wedge pressure of 60mmHg). She was assigned an ASA grade of III. There were significant anaesthetic concerns given the degree of cardiopulmonary disease; perioperative mortality risk according to the P-POSSUM risk calculator [2] was around 19%, with a morbidity risk of 70.7%. The patient, however, had a good overall quality of life and was keen on pursuing curative surgery for her malignant disease. Following adequate counselling involving the surgical and anaesthetic teams, as well as the patient and her family, the shared decision was made to proceed with surgery under combined spinal and epidural anaesthesia.

A similar technique to those already described was adopted; the L3-L4 level was used for the intrathecal injection, with the epidural catheter being sited slightly lower than in previous cases at L1. The intrathecal injection consisted of 3 mL of 0.5% heavy bupivacaine and 200 mcg of diamorphine. One intra-operative bolus of 5 mL of 0.5% bupivacaine was provided via the epidural route, followed by continuous infusion of 0.125% bupivacaine and 2 mcg/mL fentanyl for postoperative analgesia. Arterial line insertion allowed for invasive blood pressure monitoring. Inotropic support was not necessary during the procedure, nor intra-operative sedation.

A midline laparotomy was performed, and an oncological right hemicolectomy was performed with high ligation of the ileocolic, right colic, and right branches of the middle colic vessels. A side-to-side stapled ileocolic anastomosis was then fashioned. No postoperative organ support or inotropic therapy was required, and the postoperative course was uneventful. The epidural catheter was removed on the third postoperative day and the patient was discharged home on the seventh day after the operation. Histological analysis of the resected colon demonstrated an R0 resection of T2N1 (involvement of two local lymph nodes) moderately differentiated adenocarcinoma.

The patient demographics, diagnosis, imaging results, and operative interventions are summarised in Table 1.

**Table 1:** Summary of patient demographics, diagnoses, and operative interventions

Case	Age	Gender	ASA Grade	Imaging Results	Diagnosis	Surgery Performed
1	75	M	3	CT Abdomen and Pelvis - ‘Distension of the jejunum and proximal ileum down to a transition point in the right iliac fossa… terminal ileum appears swollen and oedematous. Another transition point is seen proximal to the ileocaecal junction, raising suspicions of an adhesive band (Figure 1)	Adhesional small bowel obstruction	Adhesiolysis
2	78	F	2	CT Abdomen and Pelvis – ‘ features of high-grade small bowel obstruction with multiple loops of distended small bowel… an abrupt transition towards the right iliac fossa, with angulation and twisting of bowel loops’ (Figure 2)	Adhesional small bowel obstruction	Adhesiolysis + small bowel resection with primary anastomosis
3	80	F	3	CT Thorax, Abdomen and Pelvis – ‘Focal caecal wall thickening with mesenteric ground glass changes and 4 prominent local lymph nodes’ (Figure 3)	T2 N1 M0 caecal adenocarcinoma	Right hemicolectomy with ileocolic anastomosis

## Discussion

The successful outcomes of the patients included in the case series, and the novelty of the use of locoregional anaesthesia for laparotomy locally prompted the authors to review the literature to ascertain how frequently the technique was being used elsewhere. This revealed a deficiency in studies appraising the method of delivering anaesthesia in the setting of laparotomy, with no substantial review articles identified that appraised the existing evidence base. This is, therefore, an area in which an assessment and summary of existing evidence via the means of a complete literature search would provide value. Plans for such a study were devised and are described below. 

Methods and search strategy

A formal literature search of relevant publications was performed on the 7th of January 2023 using OVID Medline, to include all PubMed-indexed works, as well as those not yet in print. No limits were imposed in terms of publication type, date, or language. The keywords used in the search were ‘laparotomy’, ‘spinal anaesthesia’ ‘epidural anaesthesia’, and ‘locoregional anaesthesia’. Any studies either discussing approaches to laparotomy without general anaesthesia, or case reports of at least one laparotomy under regional anaesthesia (spinal, epidural, or a combined spinal and epidural approach), were considered relevant, with exceptions being materials focussing solely on paediatric or non-human populations. The initial search strategy identified n=70 papers meeting the search criteria. The full search strategy is included below in Table 2.

**Table 2:** Search strategy MH = MeSH term. * = Truncated. AND, OR = Boolean operators

Search Number	Search Content	Results
S1	Laparotomy MH	20,108
S2	Epidural MH	55,200
S3	Anaesthesia, spinal MH	12,977
S4	S1 AND S2	410
S5	S1 AND S3	142
S6	S1	512
S7	S6 NOT ‘children’ MH	488
S8	S7 NOT ‘animal’ MH	48
Additional Searches		
S9	‘awake laparotomy’ NOT animal MH	9
S10	Laparotomy MH AND ‘locoregional*’	13
S11	S8 + S9 + S10	70

Publications meeting the inclusion criteria underwent abstract screening for relevance to the study question. Works in which the abstract mentioned only laparoscopic surgery were excluded, alongside studies discussing the use of regional blocks for post-operative analgesia, rather than as the sole anaesthetic measure. The remaining n=12 studies underwent full-text review and were assessed for their relevance. A further seven studies were excluded due to providing no information directly on the topic of interest once the full text was analysed.

Further manual searching of the reference sections of the five included papers was performed to identify any studies that were not detected by the search strategy. This bibliographic review returned two further studies fit for inclusion.

A summary of all studies identified by the literature search is presented in Table 3.

**Table 3:** Summary of studies identified by literature review

Authors	Location	Study Type	Method of Anaesthesia	Number of Subjects
Rodriguez et al., 2013 [5]	Spain	Case Report	Spinal	1
Consani et al., 2013 [6]	Italy	Case Report	Epidural	1
Romanzi et al., 2019 [7]	Italy	Case Series	Varied	8
Romanzi et al., 2020 [8]	Italy	Case Series	Combined Spinal-Epidural	13
Basar et al., 2017 [9]	UK	Prospective service evaluation	Spinal	21
Farda et al., 2021 [10]	Afghanistan	Case Series	Spinal	196
Pereira et al., 2021 (*no full text available)* [11]	Not stated	Case Report	Spinal	1
N.B - non-indexed studies are included in italics				

Literature review and discussion

Concerns regarding the respiratory function of the patient are the most prevalent indication in the literature for consideration of regional anaesthesia in the setting of surgery requiring laparotomy; patients with significant underlying respiratory disease have a far greater risk of requiring prolonged post-operative ventilation following general anaesthesia. The works of Rodriguez et al. [5] and Consani et al. [6] describe two cases in which significant underlying respiratory conditions prompted proceeding with laparotomy under regional anaesthesia with successful outcomes. It should be noted that there was significant variance in the anaesthetic procedures used in the two cases, with one performed purely under a spinal anaesthetic, and the other under epidural anaesthesia alone. The two cases differed in the degree of urgency of the operation, with one describing urgent exploratory laparotomy and the other a less pressing scenario of an expedited gastrectomy. In conjunction, the two provide evidence of the feasibility of regional anaesthesia for laparotomy for a range of indications, including acute emergencies, and imply that multiple options are feasible in terms of the anaesthetic protocol, depending on the scenario and experiences of the anaesthetist.

Given the wide variation between approaches put forward in the case reports, the two publications by Romanzi et al. [7,8] represent the most significant work on the topic, as the two largest, peer-reviewed studies. The authors present two separate case series both exploring the feasibility of awake laparotomy under locoregional anaesthesia, with the paper published in 2020 representing a larger continuation of the first. They present continuity in terms of patient characteristics and case selection and, therefore, allow for greater generalisability of the findings. All patients included in both studies required urgent laparotomy and had ASA scores of three or above. Respiratory comorbidities were the most commonly cited concern regarding the suitability of general anaesthesia. The majority of patients in the two series underwent combined spinal and epidural anaesthesia, along with additional sedation. Operative outcomes and postoperative pain scores were all favourable and conversion to general anaesthesia occurred in only 1/21 patients.

The available literature when considered as a whole suggests that locoregional anaesthesia for laparotomy is both feasible and can be performed with successful outcomes; however, it appears to be underutilised in the generality of practice. The technique can allow patients who would otherwise have been unsuitable for a general anaesthetic to undergo safe and effective operative intervention. There is the potential that expanding the provision of this approach may offer benefits in terms of reducing intensive care admission, by allowing for those who may well otherwise require mechanical ventilation post-operatively to avoid this.

Our case series supports the existing body of evidence in the literature regarding the feasibility of locoregional anaesthesia for laparotomy, particularly with a combined spinal-epidural approach. Our first two cases show the application of this approach in the emergency setting for patients who would have otherwise been deemed inappropriately high risk for surgery. The final case describes an elective cancer resection performed under regional anaesthesia due to the significant risk associated with subjecting this patient to general anaesthesia. The success of these patients’ surgery in terms of overall outcomes and recovery supports the need for raised awareness of the combined spinal-epidural approach as an anaesthetic tool for laparotomy. The patients included in this series have by no means a unique set of cardio-respiratory backgrounds, and there may be a wide range of patients who would be deemed high risk for general anaesthesia, but for whom surgery could become a viable option with this approach. Jaitly et al. [12] succinctly describe regional anaesthesia as suitable for those ‘living at the edge of their cardiovascular and respiratory physiological reserves’. 

There remains no standardisation of technique adopted for this anaesthetic approach to laparotomy; the existing literature shows variance likely simply according to the preference and experiences of anaesthetists happy to adopt a locoregional approach; this was also the case at our centre. The described case series will therefore be of interest to clinicians considering this method, as it offers a further description of the techniques utilised in our practice.

On a similar note, the selection of patients for this approach still remains individualised given the wide range of indications for laparotomy; there is significant variability in the surgical interventions and individual patient characteristics leading to the choice of approach. Given the lack of evidence highlighted by the literature review, the non-peer-reviewed works of Basar et al. [9] and Farda et al. [10] are of potential interest despite their limitations, as they describe other approaches to regional anaesthesia in the setting of laparotomy. 

It is worth considering whether there is the potential for stratification of patients to locoregional anaesthesia using any of the aforementioned scoring systems that assess pre-operative performance status and provide risk statistics, to guide decision-making as to whom such an approach would be preferential over general anaesthesia. Of the peer-reviewed works, the only common denominator was an ASA score of 3 or above. This is a cohort that has been shown to be at higher risk of postoperative respiratory failure with general anaesthesia [13]; however, this encompasses a wide range of patient demographics and underlying conditions. Further studies presenting more data in terms of specific comorbidity within this ASA grading and subsequent risk assessment in combination with outcomes would be of value to allow more evidence-based discussions about the appropriateness of the method on an individual basis. It is recognised that the approach will only be suitable for certain patient subsets; an important caveat from our case series is that the regional technique was only appropriate given that the bowel had not yet perforated. Viscous perforation and peritoneal contamination would contraindicate regional anesthesia both due to the intra-abdominal infection and the potential instability of the patient. 

A locoregional anaesthetic approach to laparotomy also has the potential to offer benefits outside of providing surgical and anaesthetic options to a wider range of patients. The neuromuscular blockade and subsequent requirement for mechanical ventilation in general anaesthesia have been shown to cause impairment of the respiratory system and result in an increased risk of postoperative pulmonary complications and prolonged hospitalisation [14]. Regional anaesthesia can cause a moderate degree of respiratory impairment if a high spinal block is adopted, and even lower epidural anaesthesia can reduce tidal volume and vital capacity in those with underlying lung disease [15]; however, this is less significant than with general anaesthesia. The need for level II or III care for such patients may, therefore, potentially be reduced with a locoregional approach.

This would not be the case with the rationale behind a locoregional approach studied in our work, as those in whom general anaesthesia is considered significantly high risk are more likely to have a prolonged and complex recovery simply due to their level of comorbidities and baseline function. However, one of the conclusions of the work of Romanzi et al. [7,8] was the potential for a locoregional approach to have other possible benefits. The authors discuss the possibility of the avoidance of general anaesthesia in fit patients to also facilitate a reduced need for post-operative intensive care admission, given the reduction in the ventilatory support requirements the technique offers. The authors’ conclusions were specifically in relation to mediating reduced bed availability during the COVID-19 pandemic, with additional advocacy from the British Journal of Anaesthetics for considering locoregional as an alternative to general anaesthesia [16]. Whilst the focus of this recommendation was to avoid aerosol-generating procedures during the pandemic, the authors also describe the potential for this anaesthetic technique to ‘bypass’ recovery and reduce intensive care stays. Although COVID-19 case numbers may have fallen, the bed availability per capita within the UK remains under significant pressure [17], and thus the adoption of the anaesthetic approach discussed in this paper and its associated effect on intensive care bed utilisation should be welcomed [18].

## Conclusions

There are many areas in which further research is needed to strengthen the findings already published; our literature review reveals that research on the topic of locoregional anaesthesia for laparotomy is sparse. The non-peer-reviewed works give evidence that this practice is taking place in other countries, yet there is no substantial work appraising awake laparotomy in terms of its efficacy, its acceptability to patients, nor the criteria for adopting this method. As with all case series', publication bias is a limiting factor that must be acknowledged. However, considering the technique's novelty and the dearth of evidence on the approach to laparotomy, this should not be considered too significant a limitation, and given the overall positive outcomes and range of potential benefits, the topic certainly warrants further exploration.
